# Braiding Dynamics in Semiflexible Filament Bundles under Oscillatory Forcing

**DOI:** 10.3390/polym13132195

**Published:** 2021-07-01

**Authors:** Valentin M. Slepukhin, Alex J. Levine

**Affiliations:** 1 Department of Physics & Astronomy, University of California, Los Angeles, CA 90095, USA; valentinslepukhin@physics.ucla.edu; 2 Department of Chemistry & Biochemistry, University of California, Los Angeles, CA 90095, USA

**Keywords:** semiflexible filaments, filament bundles, topological defects

## Abstract

We examine the nonequilibrium production of topological defects—braids—in semiflexible filament bundles under cycles of compression and tension. During these cycles, the period of compression facilitates the thermally activated pair production of braid/anti-braid pairs, which then may separate when the bundle is under tension. As a result, appropriately tuned alternating periods of compression and extension should lead to the proliferation of braid defects in a bundle so that the linear density of these pairs far exceeds that expected in the thermal equilibrium. Secondly, we examine the slow extension of braided bundles under tension, showing that their end-to-end length creeps nonmonotonically under a fixed force due to braid deformation and the motion of the braid pair along the bundle. We conclude with a few speculations regarding experiments on semiflexible filament bundles and their networks.

## 1. Introduction

The mechanics of semiflexible filaments has been a subject of broad interest both for its role in the mechanics of the cytoskeleton and as a testing ground for various principles of polymer and soft condensed matter physics. One feature of biological filaments, such as F-actin and collagen (a key constituent of the extracellular matrix) is their ability to form densely cross-linked bundles. These bundles are composed of a number of nearly parallel filaments cross linked by one of a variety of specialized proteins.

Previous research has focused on the collective mechanical response of permanently cross-linked filament bundles [1,2], showing that the bundle inherits a complex, scale-dependent bending modulus due to cross linking, even though the bending mechanics of the constituent filaments is comparatively simple. In most circumstances of biological interest, cross linkers detach and attach to filament bundles and their networks. As a result, such structures acquire a viscoelastic response—their stress relaxation has a complex time dependence and these systems dissipate work not only through viscous dissipation in the surrounding fluid, but also by linker unbinding. As a result, the collective mechanical response of networks of filament bundles has a nontrivial low-frequency viscoelastic response at frequencies below a characteristic linker unbinding rate [3,4].

We explore here a particular type of stress relaxation through the production and movement of defects in cross-linked bundles. Previously, we showed that bundles support a set of topological defects—loop, braids, and dislocations [5]. The lifetime of these defects is quite long, growing with the length of the bundle, since they cannot be removed by local rearrangements of the cross linking on the bundle. Defects, however, can be produced in defect/anti-defect pairs by local rearrangements, and defect pair production is predicted to be enhanced by applied compressive loads [6]. In this article, we report on theoretical studies of defect pair production under reciprocal mechanical deformations and consider how the proliferation and motion of defects affects the force–extension relation of a bundle in constant applied force experiments. We point out that there is a range of bundle mechanical parameters that allows for a nonmonotonic extension versus time curve at a constant force.

The motion of topological defects plays a critical role in the long-time plastic deformation of crystalline solids under mechanical loading. We suggest here that the defect motion plays a similar role in the slow relaxation of bundles under load. Mechanical loading can also generate defect pair production. We first consider pair production in cycles of compression and extension of one bundle. We then examine the force–extension relation of defected bundles by examining the extension of the bundle as a function of time for fixed force.

## 2. Materials and Methods

To study the conformation of the bundle under the external load, we look at the minimal energy configuration of the *N*-filament bundle under force *F*. The energy has three contributions:

(1) $$E=-F\Delta L+\mu \ell +\sum_{i=1}^{N}\int ds\frac{\kappa_{i}}{2}{\left(\partial_{s}{\hat{t}}_{i}\right)}^{2}.$$

The first term is work of the force *F*, extending the bundle by distance ΔL. Note that, unlike in Ref. [6], we pick a positive sign for the extensive force; a compressive force takes a negative sign. The second term is the missing binding energy of the cross links. Namely, if cross links are absent on the interval of the bundle of length *ℓ*, we assume that the energy of the bundle is larger by μℓ where μ is the binding energy per unit length. Here, and throughout this work, we assume that the cross linkers are highly inextensible, so where the filaments have inappropriate spacing within the braid, the cross linkers must be missing. We do not consider more elastically compliant cross linkers here. The third term is the bending energy of all the filaments in the bundle, where ${\hat{t}}_{i}\left(s\right)$ and $\kappa_{i}$ are the tangent vector and the bending modulus of the $i^{\mathrm{th}}$ filament. The integral is over the whole bundle; however, in the minimal energy state, the part of the bundle without defects is perfectly straight, so the expression under the integral is zero at this part—only the defected part of the bundle contributes to the bending energy.

**Figure 1:**
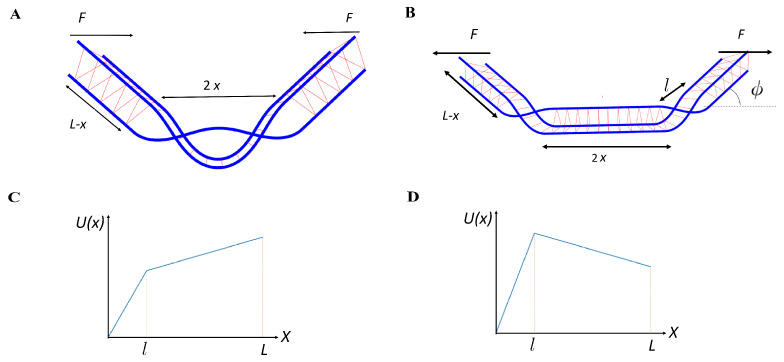
Schematic illustration: (**A**) A pair of braids produced under compression force *F* on a semiflexible filament bundle of length 2L (and comprised of three filaments), but not separated. 2x is the size of the defected region. The filaments are shown in blue, while the cross links are shown in red. (**B**) A pair of braids is now separated under applied tension. The braids produce a kink with angle ϕ and 2x is the distance between the braids (including their own size—the total excess length stored within the braid). In the lower figures, we show the piece-wise linear potential U(x) under compression (**C**) and under extension (**D**). The left part of the potential x<l corresponds to the production of defects, while the right x>l controls the separation of the defects.

In this paper, we study a three-filament bundle, and a particular type of defect: braids. Each braid can be envisioned as a permutation of the filaments within the bundle with the understanding that the product of two such permutations does not restore the bundle to its undefected state, as the filaments remain wound about each other. For a more complete description of the group of braid operations, please see Ref. [5] and the references therein. The case of a single braid, or a braid/anti-braid pair is particularly simple to analyze because, in these configurations, two filaments within the three-filament bundle remain cross-linked everywhere, while the third filament passes back and forth in between them as shown in Figure 1A,B. As a result, the equilateral triangle formed by the filaments in the plane perpendicular to their local tangent flips by π as one moves through a single braid. The anti-braid simply flips this triangle back to its original orientation. The simplicity of this structure allows one to treat the two everywhere-cross-linked filaments as a single effective filament, having twice the bending rigidity of the single filaments. Then, the problem effectively maps onto two filaments in two dimensions, one of which has a bending modulus twice as big as the other. We write the boundary conditions for this problem in terms of the tangent vector $\hat{t}$. First, we have a condition that at the ends of the braid the filament should be parallel to each other.

(2) $${\hat{t}}_{1}\left(\pm L_{1}\right)={\hat{t}}_{2}\left(\pm L_{2}\right)=\;\left(\begin{array}{c} \cos(\phi /2)\\ \sin(\phi /2) \end{array}\right).$$

where ϕ is the angle that the braid forms (see Figure 1B). Notice that here, we have also specified the direction of the coordinate axis. The second boundary condition fixes the position of the ends of the filaments:

(3) $$\int_{-L_{1}/2}^{L_{1}/2}ds{\hat{t}}_{1}\left(s\right)=\int_{-L_{2}/2}^{L_{2}/2}ds{\hat{t}}_{2}\left(s\right)+2a\cos\left(\phi /2\right)\hat{y},$$

where $L_{1,2}$ is the length of the filament in the braid, and *a* is the size of the cross-link plus two of the radii of the filament (the distance between the center lines of the cross-linked filaments). In these boundary conditions, ϕ, $L_{1}$ and $L_{2}$ are the subjects of variation. Since the braid/anti-braid pair is produced from an undeformed state, we should have a length conservation condition:

(4) $$L_{1}^{\mathrm{braid}}+L_{2}^{\mathrm{anti}-\mathrm{braid}}=L_{2}^{\mathrm{braid}}+L_{1}^{\mathrm{anti}-\mathrm{braid}}.$$

We minimize the energy Equation (1) subject to the conditions of Equations (2)–(4). The results shown here were obtained using the SciPy package in Python [7] (we performed the energy minimization with constraints (boundary conditions) using the function *minimize* with the method *trust-constr*.). We discretize each filament into 50 elements. The code is published at [8]. The numerical results coming from this discretization were previously validated by their comparison to the analytic results as shown previously in Ref. [6].

## 3. Results

### 3.1. Braid Configuration under External Force

**Figure 2:**
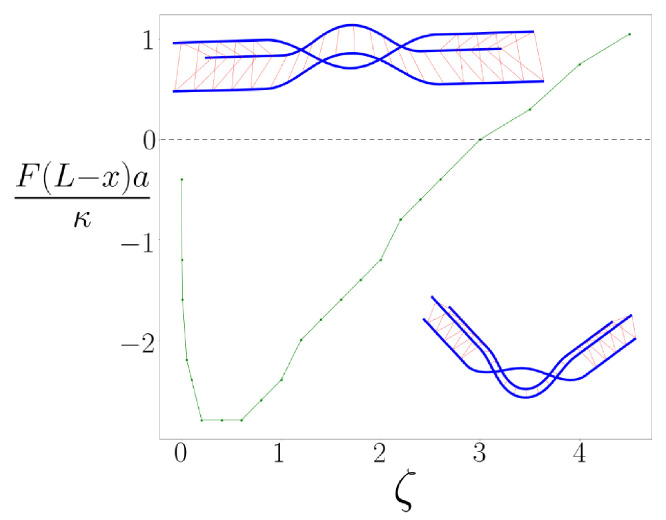
The phase boundary in the parameter space of dimensionless torque (vertical axis) and the dimensionless material parameter $\zeta =\frac{\mu a^{2}}{\kappa }$ (horizontal axis). The positive values of the torque (above the dashed line) correspond to extension, the negative (below the dashed line) to compression. Above the curve, the angle formed by the braid is zero, and below it is non-zero. The pictures represent a straight, or unkinked, braid pair (upper) and a bent, kinked braid pair (lower).

Minimizing the energy Equation (1), we obtain a phase diagram, which is shown in Figure 2 spanned by the applied torque F(L−x)a/κ and the dimensionless parameter $\zeta =\frac{\mu a^{2}}{\kappa }$ that quantifies the propensity of the braided bundle to form a kink. The kink angle is given by ϕ. Depending on ζ and the applied torque, the braid may or may not cause a localized bend of the filament or kink, as can be seen in the figure where, at large applied torques, we predict straight, braided bundles, as is intuitively reasonable. If the braids move outward along the bundle, or if we decrease the applied force, the torque acting on the kinks is reduced. In the first case, this is due to the decreasing moment arm produced by the kinked bundle, while in the second case, it is simply due to the reduced applied force.

The nonmonotonic behavior of the phase boundary is perhaps the most striking feature of the phase diagram, which was also discussed in Ref. [6]. If ζ is small, the binding energy of the linkers is also small, allowing the braid to extend along the bundle, thereby reducing its effective bending modulus. As ζ is increased, the braids become shorter and stiffer. When ζ is sufficiently large, however, the braid kinks even in the absence of an applied force in order to minimize the energy of the bundle. Because of this effect, the large-ζ limit also exhibits kinks. We note that this large-ζ limit occurs around ζ∼3, which we believe is obtainable in some biopolymer systems, including condensed DNA [9,10], cross-linked intermediate filaments [11,12] and perhaps for collagen [5,13,14,15]. We should note that we introduced the torque with the opposite sign from that used here in Ref. [6], i.e., extension is positive, and compression is negative.

**Figure 3:**
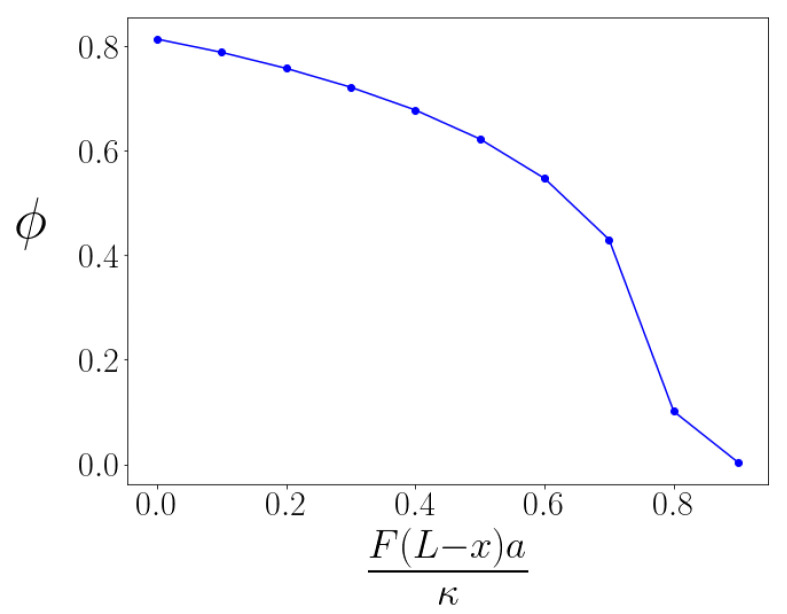
Angle produced by braid under extension as a function of the value of the torque. Dimensionless parameter ζ=4. The angle versus torque curve shows that the bending response of the braid is nonlinear, unlike the linear or Hookean response of the individual filaments.

When we consider the case of sufficiently large values of the ζ parameter, so that kinks exist even under non-zero tensile force, we may then investigate how the kink angle changes in response to that loading by determining the energy-minimized configuration of each kinked braid. As expected, the kink is straightened—the kink angle ϕ decreases—under tension. The dependence of the kink angle ϕ versus applied tension is shown in Figure 3 for a fixed ζ=4. We observe that the angle versus torque of the kinked bundle is nonlinear; the torque response of the bundle is non-Hookean. We understand this effect to occur because the structure of the energy-minimizing braid itself changes with applied torque so the braided bundle does not bend simply like an effectively thicker filament.

We will later see that the force-induced straightening of the kinks allows for the nonmonotonic behavior of the bundle’s extension versus time under a constant tensile loading. This is discussed in more detail in Section 3.4, but first, in the following section, we assume the torque to change weakly, so we can assume the kink angle to be constant, which sufficiently simplifies the study of the braid/anti-braid pair production for our analysis.

### 3.2. The Piece-Wise Linear Defect Potential and the Defect Distribution

We showed that the production of braid/anti-braid pairs in a compressed bundle can be mapped onto the Kramers escape problem in one dimension, using a single reaction coordinate [6,16]. The two defects must be first produced together in the form of defect/anti-defect pairs, which requires energy $E_{\mathrm{defect}}$. The formation of one defect introduces a length mismatch between the filaments involved that is then compensated by the second anti-defect. As a result, defect pair production entails only local rearrangements of cross linkers. During this point in the thermally-activated production of the defect pair, we may take the single reaction coordinate to represent the total length “exchanged” between defects. Once the defects separate so that a region of cross-linked bundle appears between them, the defects can no longer exchange length but they may separate along the bundle by repetitive motion. Most importantly, since the defects generate localized bends or kinks under external loading, the motion of the defects changes the end-to-end distance of the bundle under load. By separating the braids, the energy of the system decreases in response to the applied tension and increases in response to the applied compression. During this separation (under external loading) the distance between the defects plays the role of the reaction coordinate.

Taking these two aspects of the problem together, we may consider the stochastic pair production process as the thermal escape of a single fictitious particle, representing the reaction coordinate *x* in an approximately piece-wise linear potential. Before defect pair separation, x<l, where *l* is the size of the defect at the moment of the separation, the potential increases linearly as more length is exchanged between the defect pair and thus more cross links are removed. Understanding the exact form of this potential would require taking into account all different pathways leading from the properly cross-linked bundle to the bundle with braids. We make the simplest approximation, i.e., a linear potential, motivated by the fact that we need to remove the number of cross links proportional to the size of the uncross-linked region. Then, the effective potential is U(x)=Ax, with $A=E_{\mathrm{defect}}/l$. The energy of the defect incorporates the bending energy of the filaments, the missing binding energy of the cross links absent in the defected region, and the work of the applied force:

(5) $$E_{\mathrm{defect}}=E_{\mathrm{bending}}\left(\phi \right)+E_{\mathrm{binding}}\left(\phi \right)-2F\left(L-x\right)\cos\left(\phi \right)-2Fx,$$

where ϕ is the angle formed by a single braid (see Figure 1), *F* is a force acting on the ends of the bundle (positive sign is chosen for the extension), and 2L is a length of the bundle. The angle ϕ is determined by energy minimization with respect to it. As the defects separate, *x* grows. The angle also changes but as soon as x≪L, this change contributes to the energy at the next order in the small parameter x/L. We address the explicit dependence of the angle on interbraid separation in Section 3.4. Omitting this effect, we can again write an effective potential U(x)=Al+B(x−l) with B=2F(cosϕ−1). Thus, we may explore the pair production process using the following potential:

(6) $$U\left(x\right)=\;\left\{\begin{array}{c} Ax,x<l\\ Al+B(x-l),l<x<L\\ \infty ,x>L \end{array}\right.$$

Before considering dynamics, we use this potential to consider the equilibrium distribution of defects on a bundle of length *L*. Specifically, we consider the equilibrium separation of two defects. Using Equation (6), it is trivial to write the probability distribution in this potential: $p\left(x\right)=\frac{1}{Z}e^{-\beta U(x)}$ where the partition function *Z* is given by the following:

(7) $$Z=\int_{0}^{L}e^{-\beta U(x)}dx,$$

and $\beta =1/k_{B}T$ is the inverse temperature. A straightforward calculation arrives at the partition function written as the sum of two parts corresponding to the two pieces of the potential as follows:

(8) $$Z=Z_{1}+Z_{2}.$$

with

(9) $$Z_{1}=\frac{1}{\beta A}\left(1-e^{-\beta Al}\right)$$

and

(10) $$Z_{2}=\frac{1}{\beta B}e^{-\beta (A-B)l}\left(e^{-\beta Bl}-e^{-\beta BL}\right).$$

Taking the ratio of these partition sums, we obtain the ratio of observable separated braid pairs to strongly interacting and co-localized braids:

(11) $$Z_{2}/Z_{1}=\frac{Ae^{-\beta (A-B)l}\left(e^{-\beta Bl}-e^{-\beta BL}\right)}{B(1-e^{-\beta Al})}.$$

From the above, we see that in the thermal equilibrium, we expect there to be a low density of separated braids, at least at low temperatures (T≈300 K). In particular, if the thermal energy is much lower than the cross-linker binding energy (which is typically true in biopolymer filament systems) we expect an exponentially small density of braids ($\propto e^{-E_{\mathrm{braid}}\beta }$, where for known filaments $\beta E_{\mathrm{braid}}\gg 1$. In fact, it appears that the smallest value of $\beta E_{\mathrm{braid}}$ is found for DNA bundles condensed by polyvalent ions where $\beta E_{\mathrm{braid}}\approx 50$ [6,9,10]. Braid pairs, however, can be generated either during bundle formation or via cycles of compression and expansion, as would be expected in a bundle network under reciprocal shear.

### 3.3. The Nonequilibrium Braid Distribution in a Time-Dependent Potential

When one applies a time-varying force, the effective potential controlling the production and motion of the braids also changes in time. As a result, we cannot rely on the equilibrium distribution discussed in the previous section. Instead, we have to solve the Smoluchowski diffusion equation for defect density ρ(t,x):

(12) $$\partial_{t}\rho \left(t,x\right)=D\partial_{x}\left[\left(\partial_{x}-\beta F\left(t,x\right)\right)\rho \left(t,x\right)\right],$$

where the force now takes the following form:

(13) $$F\left(t,x\right)=\;\left\{\begin{array}{c} A(t),x<l\\ B(t),l<x<L. \end{array}\right.$$

During compression $A\left(t\right)=A_{c}$ and $B\left(t\right)=B_{c}$. During expansion $A\left(t\right)=A_{s}$, $B\left(t\right)=B_{s}$ (note that $B_{s}$ is negative). In the above, *D* is the defect diffusion constant. We cannot solve this equation analytically; however, we can provide a qualitative analysis. To simplify, we assume that *A* and *B* are fixed during each period of compression and expansion. We explore how the defect production rate depends on the lengths of these periods of compression and extension. We also estimate the maximal defect production rate.

The transport time from 0 to *l* in the potential is controlled by the constant *A*. This is the braid pair production rate when the braids have a stored length of *l*. This problem is simply the first passage time [16] to reach *l* in the linearly increasing potential, which we may estimate to be as follows:

(14) $$T_{0l}=\frac{1}{D}\frac{e^{\beta Al}}{\beta^{2}A^{2}}.$$

Similarly, we estimate the transport time from *L* to *l*. This gives an approximate value of the lifetime of the braid pair since when their separation returns to *l*, they will likely annihilate. Here, we must distinguish between two limiting cases. In the first case, we consider purely diffusive braid motion and in the second, we look at the deterministic transport of the braids under an applied force, using a mobility set by the diffusion constant and the Einstein relation. We find the following:

(15) $$T_{Ll}=\;\left\{\begin{array}{c} L^{2}/D,B\beta >1/L\\ L/(B\beta D),B\beta <1/L. \end{array}\right.$$

In general, where we expect there to be both diffusive and advective defect motion, we find that the time for defects to recombine is the following:

(16) $$T_{Ll}=\frac{L^{2}}{D(1+\beta BL)}.$$

In the limit of large *L*, $T_{Ll}\gg T_{0l}$ so that the time for distant defect pairs to come together and potentially annihilate is much greater than their production time. If braids are able to separate sufficiently, we expect this ordering of time scales to be valid and thus predict braid proliferation on the bundle.

Because the production time $T_{0l}$ has an exponential dependence on *A*,

(17) $$T_{0l}^{\mathrm{compression}}\ll T_{0l}^{\mathrm{stretching}},$$

since the *A* parameter is much larger under stretching than it is under compression, $A_{c}<A_{s}$. If we choose the time dependence of the applied force so that the stretching time $\tau_{s}$ and compression $\tau_{c}$ satisfy the inequalities

(18) $$T_{0l}^{\mathrm{compression}}\ll \tau_{c}\ll \tau_{s}\ll T_{0l}^{\mathrm{stretching}}\ll T_{Ll},$$

we may analyze the dynamics of the system using a few approximations.

Since the braid production time during compression is small compared to the compression time: $T_{0l}^{\mathrm{compression}}\ll \tau_{c}$, then, during the compression period, the density on the left 0<x<l equilibrates. Since $\tau_{s}\ll T_{0l}^{\mathrm{compression}}\ll T_{Ll}$, during the stretching period, the probability density ρ(x) decreases near the potential maximum at x=l, but it is highly unlikely that thermally excited hopping over the barrier at *l* occurs. Since $\tau_{c}\;\ll \;\tau_{s}\;\ll \;T_{0l}^{\mathrm{compression}}\;\ll \;T_{Ll}$, the applied force is changing sufficiently fast that the density of the right of the potential x>l may be replaced by its time-averaged value. Moreover, since $\tau_{c}\ll \tau_{s}$, density on the right of the potential is effectively determined by the dynamics during the stretching period. Because the compression period is shorter, the already produced braids are unlikely to be driven together and annihilate. As a result, we conclude that the density distribution on the left is effectively determined by the compression period and the density on the right by the stretching period. Finally, we note that the Smoluchowski diffusion equation requires the continuity of both the probability density and its current at the boundary x=l. From these conditions, we obtain a value of the averaged density on the right as a function of the equilibrium density on the left. This implies that with this sequence of inequalities, the braid production and separation may be considered to take place in a time-averaged, effective potential where the production part is set by the compression forces and braids separate under a force related to extension. Specifically, we consider the following:

(19) $$U\left(x\right)=\;\left\{\begin{array}{c} A_{c}x,x<l\\ A_{c}l+B_{s}\left(x-l\right),l<x<L\\ \infty ,x>L. \end{array}\right.$$

In this potential, the probability of braids on the left x<l will be proportional to the following:

(20) $$Z_{1}=\frac{1}{\beta A_{c}}\left(1-e^{-\beta A_{c}l}\right),$$

while on the right,

(21) $$Z_{2}=\frac{1}{\beta B_{s}}e^{-\beta (A_{c}-B_{s})l}\left(e^{-\beta B_{s}l}-e^{-\beta B_{s}L}\right).$$

The ratio is as follows:

(22) $$Z_{2}/Z_{1}\approx -\frac{A_{c}}{B_{s}}e^{-\beta (A_{c}-B_{s})l}e^{-\beta B_{s}L}\gg 1,$$

which exceeds the case of only compression by a factor of $e^{\beta |B_{s}|L}$, and the case of only expansion by a factor of $e^{\beta (A_{s}-A_{c})l}$—see Equation (11).

### 3.4. Constant Force Stretching Dynamics of a Braided Bundle

We now consider an experiment in which one stretches a previously compressed bundle (by laser tweezers or other means) at a fixed force and determines the time rate of change of the bundle’s length. This is akin to a step force rheological measurement, and is closely related to determining the force-extension curve of a filament or filament bundle. Typically, in such force extension measurements, one considers the limit of slow extension so that the observed length corresponds the thermal equilibrium prediction under a fixed force [17]. In this case, however, the extension of the bundle will be time dependent, even though the force is constant.

**Figure 4:**
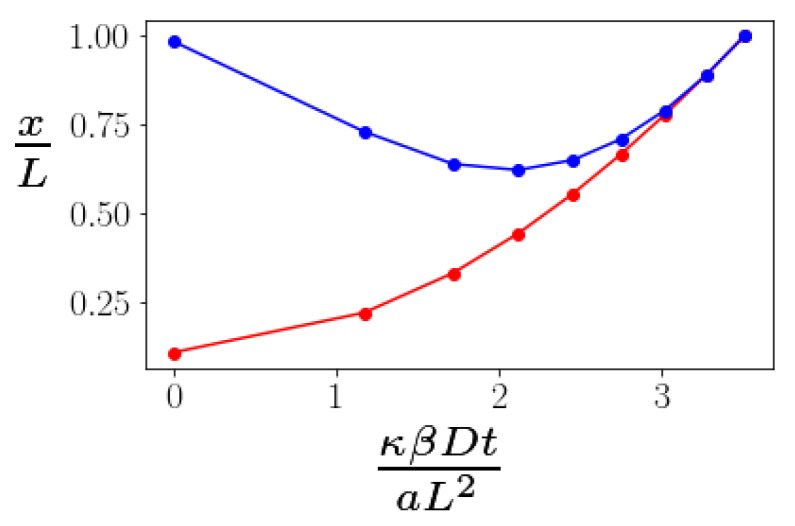
The time evolution of the distance between two defects (red) and the end-to-end distance of the bundle (blue) under a constant applied tensile force $F=0.9\frac{\kappa }{aL}$ plotted as a function of non-dimensionalized time. The cross linker binding energy is $\mu =4\frac{\kappa }{a^{2}}$.

To study this problem, we minimize the energy of the bundle under a fixed stretching force. By doing so, we assume that the bending of the kink angles at the braids is fast compared to the time scale of measurement of the end-to-end distance. We do not, however, assume that the advection and diffusion of the braids is similarly fast. Doing this energy minimization numerically, we obtain the energy of the bundle as a function of the distance between two defects. Then, the average displacement will be controlled by a drift velocity $\frac{dx}{dt}=-\beta D\frac{dE}{dx}$. Solving this equation numerically, we find time dependence shown in Figure 4. The time dependence of the bundle’s extension is nonmonotonic: as defects diffuse from each other, the bundle initially becomes shorter. This happens due to the fact that as *x* grows, the L−x becomes smaller; hence, the moment of the force decreases as well. Since the stretching torque decreases, the kinks become less stretched and their angles increase (see Figure 3), decreasing the end-to-end distance ΔL=2(x+(L−x)cosϕ).

## 4. Discussion

Semiflexible filament bundles admit three classes of topological defects: loops, braids, and dislocations. These defects are all likely to be produced in the formation of bundles from solutions of semiflexible filaments by the introduction of cross-linking agents. They are, however, unlikely to form spontaneously in thermal equilibrium when the filament bundles are also chemically equilibrated with a reservoir of cross linkers. Here, we have pointed out that the reciprocal compression and extension of filament bundles, however, is capable of producing a higher nonequilibrium density of braid defects within a bundle. The key insight is that bundles under compression can locally buckle, producing braid, anti-braid pairs. Upon subsequent extension, these braid pairs will be driven to separate, as long as they do not immediately annihilate. In order to ensure that the braid pairs produced in the previous compression cycle (and separated during the previous extension part of that cycle) do not annihilate during the subsequent compressive cycle, one needs to introduce an asymmetry between the period of compression (short) and the period of extension (long). Other cycles of compression and extension will also produce braid, anti-braid pairs, but at lower density. We predict that the short compression period following the long extension one will result in the maximum possible defect density.

We also examined the extensional dynamics under a fixed tensile load of semiflexible bundles containing a braid defect pair. Here, one does not observe the standard worm-like chain force extension relation at very low frequencies. The extension of the bundle is not controlled by the depletion of the length reservoir associated with the thermally-generated undulations of the bundle, but rather by the motion of the braid defects and the bending of the kinks associated with those defects. In essence, our predictions refer to the analog of plastic deformation in solids associated with defect motion rather than the (entropic) elastic response of the bundle, which, due to cross linking, is suppressed. We note that the end-to-end distance of the bundle varies nonmonotonically with time under a constant tensile load. This somewhat counter-intuitive result occurs due to the combination of two effects: braid separation, which lengthens the bundle, and kink angle relaxation, which shortens it.

For experimental verification of these predictions, there is no more direct measurement than compression/extension experiments on individual semiflexible filament bundles using laser or magnetic tweezers to manipulate the bundle’s stress state [18]. In addition, one expects that the imagining experiments on compressed semiflexible filament bundles should produce observable kinks (localized bending defects) rather than the uniform curvature of the entire bundle, as would be expected from classical Euler buckling. Although less direct in testing the predictions made here, standard shear measurements and studies of stress relaxation in networks of filament bundles with transient cross linkers at long times or low frequencies are particularly relevant to the present work. We imagine that, at sufficiently long times, stress relaxation will be dominated by plastic deformation of the network, comprised of both tearing and reattachment of bundles from each other, and the plastic deformation of the individual bundles themselves, presumably following the mechanisms discussed here. We do not, as yet, understand how to distinguish these dynamics in rheological data, and this remains one of the principal open questions related to this work.

Other open questions involve the mechanical compliance of the cross linkers and their binding kinetics. In this work, we considered the cross linkers to be essentially inextensible so that they must fall off the defected regions where the inter-filament spacing is no longer equal to the cross linkers’ length. We imagine that cross linker redistribution and defect energies will be affected by the elastic compliance of the cross linkers. Previous studies of the peeling of bead-spring models of filaments [19,20] and of semiflexible filaments [21] bonded to substrates by cross linkers have demonstrated the importance of the cross linkers’ compliance on the unbinding dynamics. Based on these studies, we anticipate that the inclusion of cross linker compliance may affect both the kinetics of defect motion and their creation/annihilation rates. For example, sufficiently elastic cross linkers may remain in the core of the defects incurring a certain energy of deformation. This will clearly change the defect energies. In fact, sufficiently compliant cross linkers may even allow for Euler buckling of the bundle under compression. Lastly, we point out that we have assumed that cross linkers are able to bind and unbind on short time scales, compared to the observation time for both defect pair production and motion along the bundle. When investigating bundle mechanics at sufficiently short time scales, one must consider the possibility that the cross linker distribution is no longer in equilibrium with the bundle in its current stress state. We leave these open questions to future work.

## Data Availability

Not applicable.
